# Association Between Dietary Patterns and Fluorosis in Guizhou, China

**DOI:** 10.3389/fnut.2019.00189

**Published:** 2020-01-21

**Authors:** Jun Liu, Sheng Yang, Ming-jiang Luo, Ting Chen, Xiao-juan Ma, Na Tao, Xun Zhao, Dong-hong Wang

**Affiliations:** ^1^Experimental Teaching Demonstration Center for Preventive Medicine of Guizhou Province, Zunyi Medical University, Zunyi, China; ^2^Department of Pharmacy, Affiliated Hospital of Zunyi Medical University, Zunyi, China; ^3^Department of Chronic Disease, Center of Disease Control and Prevention of Zhijin County, Zhijin, China; ^4^Department of Gynaecology and Obstetrics, Affiliated Hospital of Zunyi Medical University, Zunyi, China

**Keywords:** dietary pattern, factor analysis, fluorosis, coal burning, case-control and matched study

## Abstract

**Objective:** Many studies have explored the effects of individual foods or nutrients on fluorosis, but no studies have focused on dietary patterns. This study examined the relationship between dietary patterns and coal-burning fluorosis in Guizhou, China.

**Methods:** This 1:1 matched case-control study was conducted in Zhijin County of Guizhou province with a sample size of 200 cases of fluorosis and 200 age and gender matched controls. Habitual dietary intake was assessed by face-to-face interviews, using a validated 75-item food frequency questionnaire (FFQ) and various covariates using structured questionnaires. The dietary patterns were identified by factor analysis.

**Results:** The factor analysis identified three major dietary patterns which were labeled healthy, easy-to-roast and high protein. After adjusting for various confounding factors, a decreased risk for fluorosis was observed in the highest tertile of the healthy dietary pattern relative to the lowest tertile (OR = 0.47, 95% CI = 0.27–0.84, P-trend = 0.003) and a positive association was observed between the easy-to-roast dietary pattern and fluorosis risk (OR = 2.05, 95% CI = 1.15–3.66), with a significant linear trend (*P* = 0.017). We did not find an association between fluorosis risk and the high protein dietary pattern. The relationships remained significant when the analyses were stratified by gender and fluorosis subtypes.

**Conclusion:** The healthy dietary pattern may lower coal-burning fluorosis risk; in contrast, the easy-to-roast dietary pattern significantly increases the risk of coal-burning fluorosis.

## Introduction

Endemic fluorosis is a disease affecting mainly the bones and teeth, caused by exposure to high levels of fluorine in the environment. Over 70 million people may be affected globally, including China, India, Africa and South America. Reports predicted that over 26 million people in China suffer from fluorosis due to fluoride contamination in their drinking-water. A further 16.5 million people burn fluoride-rich coal indoors for heating and cooking, a process which is termed coal-burning fluorosis (CBF) (1). The most severe CBF case was located in Guizhou, China; 43% of its districts and counties had fluorosis incidents (2). Although CBF cases have been recently reduced through the use of improved stoves and awareness education, the incidence rate of fluorosis remains high (3, 4). This disease seriously restricts economic development and reduces quality of life. Therefore, further strategies are needed to prevent and control fluorosis.

Nutrition may play an important role in the development of fluorosis. Studies have not been successful at establishing a consistent association between CBF risk and specific foods and nutrients [e.g., corn (5, 6) chili (6), sour soup (7), vitamins (8–11), and calcium (12, 13)]. The inconsistent results linking foods and fluorosis may, in part, reflect the difficulty of disentangling the high degree of correlation among dietary constituents. To address this problem, Schwerin et al. proposed using dietary patterns to examine diet-disease relationships (14). Dietary patterns incorporate food interactions in the diet and consider the overall diet, reflecting more closely real-world food intakes, and are therefore more useful to predict disease risk than individual foods or nutrients (15). Factor analysis is a variable consolidation technique that can be applied to dietary data to identify underlying dietary patterns based on the inter-correlations of food groups. To our knowledge, there have been no studies on the association between dietary patterns and fluorosis. Therefore, we conducted this 1:1 matched case–control study to identify major dietary patterns among Chinese residents of Guizhou province and to examine the associations of these dietary patterns with the risk of fluorosis.

## Subjects and Methods

### Study Subjects

This 1:1 matched case-control study was conducted between July and August 2015 in Zhijin County, Guizhou Province, China, where coal-burning fluorosis is endemic. All of the CBF cases were diagnosed by the Zhijin County Disease Control and Prevention Center according to the Chinese Diagnostic Criteria of Dental Fluorosis (WS/T208-2011, China) and the Chinese Diagnostic Criteria of Endemic Skeletal Fluorosis (WS/T 192-2008, China). Cases were identified by one professional doctor in Zhijin Center for Disease Control and Prevention, and eligible cases, aged 18–75 years old, were recruited through village doctors. Subjects in the following situations were excluded: (1) pathological fractures or fractures from car accidents, falls and other fractures caused by violence; (2) patients with tetracycline stained teeth and dental cavities; (3) patients previously diagnosed with cancer, coronary heart disease, stroke, gout, or kidney disease; (4) patients who self-reported substantial changes in dietary habit within the previous 5 years. For each CBF case, a control participant was selected from residents living in the same county and within the same sex and age group (±3 years). Control participants were required to be at least 18 years old, to have lived in Zhijin County for at least the previous 10 years and were selected according to the same exclusion criteria as CBF patients. The study sample consisted of 200 CBF patients and 200 controls. All of the study participants signed informed consent forms prior to the interview and the Medical Ethics Committee of Zunyi Medical University approved the project (No. 2014-1-003).

### Data Collection

The following information was collected by trained interviewers through face-to-face interviews using a structured questionnaire. The questionnaire included (1) socio-demographic characteristics (age, gender, education level); (2) lifestyle habits (alcohol drinking, tea drinking, smoking status, domestic fuel type, use of improved stoves, consumption of roasted food); (3) dietary habits in the year before the interview; and (4) relevant diseases (hypertension, diabetes, gout, kidney disease, cancer, heart-related diseases, and stroke). Tea drinkers were defined as individuals who drank tea at least twice weekly; Likely, smokers are the participants who smoked at least five packs of cigarettes a year and those who drank alcohol at least once a week continuously for at least 6 months were considered alcohol drinkers. CBF patients and controls were required to respond to the same questionnaire.

### Dietary Assessment

The participants were instructed to recall their usual dietary consumption using a validated 75-items food frequency questionnaire (FFQ) (16). Foods consumption was estimated in terms of the frequency of use per day, per week, per month, or per year. To help the participants quantify the amounts, they were provided with color pictures of foods with approximate proportion sizes. The selected value for each food was then converted to reflect the daily intake. Energy and nutrient intakes were computed based on the China Food Composition database (17).

### Food Grouping and Identification of Dietary Patterns

To reduce the complexity of the data, 75 food items from the FFQ were categorized into 25 predefined food groups according to similarities in their nutrient profiles or processing methods. Dietary factor patterns were then identified by principal component analysis. A varimax (orthogonal) rotation was performed to improve interpretability and minimize the correlation. Factors were retained based on eigenvalues (>1.3 cut-off), scree plots and factor interpretability (18). Factor loadings for each food group were calculated and factor scores per pattern were calculated for each subject by summing the total intake of each food group, weighted by their factor loadings. High scores represented a high intake of food items resulting in a positive loading on the corresponding dietary pattern, whereas low scores represented a low intake of those items. Food groups with absolute values >0.25 are presented in Table 1. Based on the analysis, three categories were selected.

**Table 1:** Characteristics of fluorosis cases and controls.

**Variables**	**Case** ** (*n* = 200)**	**Control** ** (*n* = 200)**	***P*-value**
Age, years (Mean ± SD)	49.8 ± 13.4	49.7 ± 13.5	0.916
Body mass index, kg/m^2^ (Mean ± SD)	23.8 ± 3.9	23.6 ± 3.9	0.253
Urine fluoride, mg/L (Mean ± SD)	1.62 ± 1.0	1.28 ± 0.6	<0.001
Duration of residence, years (Mean ± SD)	39.7 ± 18.1	39.717.1	0.951
Dental fluorosis	193	193	
Skeletal fluorosis	87	87	
Female (%)	115 (57.5)	115 (57.5)	0.923
Ethnic (%)			0.313
Han	118 (59.0)	108 (54.0)	
Others	82 (41.0)	92 (46.0)	
Marital status (%)			0.584
Married or cohabitation	172 (86.0)	168 (84.0)	
Divorce or widowed	18 (9.0)	17 (8.5)	
Unmarried	10 (5.0)	15 (7.5)	
Education level, years (%)			0.008
≤6	162 (81.0)	141 (70.5)	
6–9	32 (16.0)	39 (19.5)	
≥10	6 (3.0)	20 (10.0)	
Household income, Yuan/month (%)			0.049
<500	23 (11.5)	11 (5.5)	
500–2,000	55 (27.5)	64 (32.0)	
2,000–6,000	94 (47.0)	86 (43.0)	
≥6,000	28 (14.0)	39 (19.5)	
Fuel type (%)			0.053
Raw coal	116 (58.0)	127 (63.5)	
Mixed coal	44 (22.0)	33 (16.5)	
Firewood	40 (20.0)	40 (20.0)	
Smoker^‡^(%)	74 (37.0)	72 (36.0)	0.835
Alcohol drinker^§^ (%)	66 (33.0)	61 (30.5)	0.568
Tea drinker^||^ (%)	83 (41.5)	74 (37.0)	0.357
Like roasting food^#^ (%)	118 (59.0)	100 (50.0)	0.070
Improved stove use^¶^ (%)	149 (74.5)	171 (85.5)	0.006

‡ Smokers were defined as having smoked at least one cigarette daily for at least 6 consecutive months.

§ *Alcohol drinker was defined as having had wine (beer, white wine and red wine) at least once per week for at least 6 consecutive months*.

|| *Tea drinkers were defined having drank tea at least twice weekly*.

¶ *Improving the kitchen stove to exclude the fluoride out of the room to decrease the pollution of the air indoors*.

# *A habit of roasting food with indoor burning of fluoride-rich coal*.

### Urinary Fluoride Detection

A 10 ml urine sample was collected from each participant. Urinary fluoride concentration was analyzed by a standardized method (WS/T 89-2015, China). Briefly, 1 ml of urine was mixed with 24 ml of deionized water, one pill of fluoride adjustment buffer powder was added to each 25 ml mixture. Then, the concentration of urine fluoride in the mixture was measured by a HQ40d portable meter and the concentration of fluorine in the urine of the subjects was calculated according to the corresponding proportion. The coefficient of variation for the urinary fluoride was 2.29%. The urine fluoride concentrations for all participants were categorized into either the normal category (<1.6 mg/L) or the abnormal category (more than 1.6 mg/L), according to national criteria (WS/T 256-2005).

### Statistical Analysis

The data were tabulated as mean and SDs for continuous variables, or by proportions for categorical variables. To achieve an approximately normal distribution for statistical analysis, a logarithmic transformation and a square root transformation were performed for intake of energy and dietary nutrients, respectively. Dietary nutrient intake data were controlled for total energy intake by the regression residual method (19). The chi-square test and *t*-test were used to test the differences in socio-demographics and nutrient intakes of the fluorosis cases and controls. The factor scores were categorized into tertiles. T1, T2, and T3 represented a low, medium and high food intake patterns, respectively.

Conditional logistic regression analysis models were constructed to obtain odd ratios (ORs) and corresponding 95% confidence intervals (95% CIs) as estimates of relative risk, which are shown for the second and third tertiles, with the lowest tertile group as the reference category. We estimated the risk of fluorosis associated with each of the three dietary patterns separately. In the multivariate models, the relationships between dietary patterns and the risk of fluorosis were further examined after adjusting for various potential confounding variables such as duration of residence in Zhijin, ethnicity, marital status, education level, household income, body mass index (BMI), urinary fluoride level, smoking, alcohol drinking, tea drinking, domestic fuel type, and use of improved stove, whether, or not like roasting food. We further examined the association between dietary pattern and CBF across different subgroups stratified by gender (man/women) and whether or not like roasting food (Yes/No). To test the *p*-value of the trends, the ordinal values of the tertile of each factor score were entered as continuous variables in a logistic regression analysis model. In this study, SPSS 18.0 was used for the statistical analysis, all of the *P*-values were two sided and statistical significance was determined at the *p* < 0.05 level.

## Results

The characteristics of the CBF cases and controls are presented in Table 1. Of the 200 CBF patient–control pairs, 193 patient pairs had dental fluorosis and 85 patient pairs had skeletal fluorosis. The average age was 49.8 years in the CBF patients and 49.7 years in the control group. The CBF group had lower education levels, household incomes, higher urinary fluoride levels, and were less likely to use an improved stove. No significant differences were found between the CBF cases and control in marital status, BMI, duration of residence in Zhijin, smoking status, alcohol, or tea drinking.

We identified three major dietary patterns using the factor analysis procedure, presented in Table 2. The first pattern, named the high protein dietary pattern, was characterized by a high intake of beef, seafood, poultry, fish and pork. The second pattern, named the healthy dietary pattern, was characterized by a high intake of fruit, dairy, legume, nuts, and fungus. The third pattern was characterized by a high intake of squash, potatoes, corn, dried chili and cured meat. This last group was labeled as the easy-to-roast dietary pattern, because Zhijin County residents commonly roast this last group of foods before consumption. Dietary patterns 1, 2, and 3 accounted for 7.9, 7.1, and 5.7% of the variability, respectively, and 26.7% of the variance in food intake overall.

**Table 2:** Factor-loading matrix for the three major dietary patterns^*^.

**Food items**	**High protein pattern** ** (Factor 1)**	**Healthy pattern** ** (Factor 2)**	**Easy-to-roast food pattern** ** (Factor 3)**
Beef	0.690	–	–
Sea food	0.665	–	–
Pork	0.521	–	–
Poultry	0.622	–	–
Fish	0.551	–	–
Dairy	–	0.558	–
Legume	0.336	0.557	–
Sour soup	–	0.496	–
Dark-colored fruits	–	0.565	0.284
Light-colored fruits	–	0.573	–
Nut	–	0.440	–
Mushroom and algae	–	0.408	–
Gourds	0.258		0.562
Corn	–		0.496
Dried chili	–		0.474
Potato	–		0.555
Cured meat	–		0.465
Dark-colored vegetables	0.287		–
Light-colored vegetables	–		0.270
Houttuynia cordate	0.316		–
Eggs	0.271		–
Cooking oil	–	0.296	–
Root vegetables	–	0.259	–
Cereals	–		0.322
snacks	–	0.306	–

* *Absolute values <0.25 were excluded for simplicity*.

As shown in Table 3, univariate analyses showed CBF was inversely associated with the healthy dietary pattern (*p* < 0.001) but positively associated with the easy-to-roast dietary pattern. A significant relationship remained even after adjustments were made for duration of residence in Zhijin, ethnicity, marital status, education level, household income, smoking status, alcohol drinking, tea drinking, daily energy intake, dietary supplement usage, domestic fuel type, use of improved stoves, and whether or not like roasting food. The risk of CBF decreased by approximately 53% in the highest tertile of the healthy dietary pattern group, compared with the reference lowest tertile (OR = 0.47, 95% CI = 0.27–0.84, *p* = 0.003). Significant relationships were observed between CBF risk and the healthy dietary pattern (T3 vs. T1: OR 2.05, 95% CI 1.15–3.66, *p* = 0.017). In addition, there was no association between the high protein diet pattern and CBF.

**Table 3:** Odds ratios (ORs) and 95% confidence intervals (95% CIs) of fluorosis according to tertiles of factor scores for each dietary pattern.

	**Tertiles of factor scores for dietary pattern**			***P*-trend**
	**Tertile 1^§^**	**Tertile 2**	**Tertile 3**	
**High protein pattern**				
Case/control	57/65	83/67	60/68	
OR (95% CI)^1^	1	1.44 (0.88, 2.34)	1.02 (0.59, 1.74)	0.944
OR (95% CI)^2^	1	1.62 (0.94, 2.76)	1.47 (0.81, 2.65)	0.205
**Healthy pattern**				
Case/control	112/66	48/66	40/68	
OR (95% CI)^1^	1	0.42 (0.25, 0.69)^*^	0.33 (0.19, 0.57)^**^	<0.001
OR (95% CI)^2^	1	0.42 (0.24, 0.71)^*^	0.47 (0.27, 0.84)^*^	0.003
**Easy-to-roast pattern**				
Case/control	37/65	70/66	93/69	
OR (95% CI)^1^	1	1.91 (1.12, 3.25)^**^	2.49 (1.45, 4.30)^**^	0.001
OR (95% CI)^2^	1	1.75 (0.99, 3.09)	2.05 (1.15, 3.66)^*^	0.017

*OR, odds ratio; CI, confidence interval*.

§ *Tertile1 was the reference tertile*.

1 *Model 1 Crude adjusted ORs were obtained without further adjustment of covariates*.

2 *Model 2 adjusted for duration of residence in Zhijin, ethnic, marital status, education level, body mass index, urinary fluoride level, household income, smoking, alcohol drinking, tea drinking, daily energy intake, supplement use, fuel type, improved stove use, like roasting food*,

* *P < 0.05*,

** *P < 0.01*.

The results of dental and skeletal fluorosis sub-type analyses are shown in Table 4. We observed a similar dental fluorosis incidence in the healthy diet group as in the easy-to-roast diet group. Skeletal fluorosis was negatively associated with the healthy diet group (*p* = 0.025), but marginally positively associated with the easy-to-roast diet group after adjustment (*p* = 0.089). Stratified analyses showed a negative association between the healthy dietary pattern and CBF in women but not in men, and a positive association between the easy-to-roast dietary pattern and CBF (Supplementary Table 1).

**Table 4:** Odds ratios (ORs) and 95% confidence intervals (95% CIs) of dental and skeletal fluorosis according to tertiles of factor scores for each dietary pattern.

	**Tertiles of factor scores for dietary pattern**			***P*-trend**
	**Tertile 1^§^**	**Tertile 2**	**Tertile 3**	
**DENTAL FLUOROSIS**				
**High protein pattern**				
Case/control	65/63	71/64	57/66	
OR (95% CI)^1^	1	1.40 (0.85, 2.31)	0.95 (0.55, 1.62)	0.821
OR (95% CI)^2^	1	1.49 (0.85, 2.63)	1.30 (0.70, 2.42)	0.428
**Healthy pattern**				
Case/control	110/64	47/64	36/65	
OR (95% CI)^1^	1	0.42 (0.25, 0.70)^*^	0.32 (0.18, 0.55)^**^	<0.001
OR (95% CI)^2^	1	0.45 (0.25, 0.81)^*^	0.46 (0.25, 0.84)^*^	0.001
**Easy-to-roast pattern**				
Case/control	38/63	68/65	87/65	
OR (95% CI)^1^	1	1.89 (1.09, 3.06)^**^	2.45 (1.39, 4.28)^**^	0.002
OR (95% CI)^2^	1	1.70 (0.92, 3.12)	2.03 (1.11, 3.72)^*^	0.023
**SKELETAL FLUOROSIS**				
**High protein pattern**				
Case/control	29/29	31/26	27/32	
OR (95% CI)^1^	1	1.00 (0.41, 2.42)	0.65 (0.27, 1.56)	0.326
OR (95% CI)^2^	1	1.38 (0.63, 3.02)	1.19 (0.51, 2.80)	0.646
**Healthy pattern**				
Case/control	50/32	21/29	16/26	
OR (95% CI)^1^	1	0.49 (0.23, 1.07)	0.34 (0.14, 0.84)^*^	0.007
OR (95% CI)^2^	1	0.51 (0.24, 1.08)^*^	0.41 (0.18, 0.93)^*^	0.021
**Easy-to-roast pattern**				
Case/control	15/30	32/26	40/31	
OR (95% CI)^1^	1	2.31 (0.99, 5.39)	2.59 (1.09, 6.17)	0.039
OR (95% CI)^2^	1	2.36 (1.01, 5.50)^*^	2.29 (1.01, 5.19)^*^	0.036

*OR, odds ratio; CI, confidence interval*.

§ *Tertile1 was the reference tertile*.

1 *Model 1 Crude adjusted ORs were obtained without further adjustment of covariates*.

2 *Model 2 adjusted for duration of residence in Zhijin, ethnic, marital status, education level, body mass index, urinary fluoride level, household income, smoking, alcohol drinking, tea drinking, fuel type, improved stove use, like roasting food*,

* *P < 0.05*,

** *P < 0.01*.

## Discussion

To our knowledge, this matched case-control study was the first to use factor analysis to evaluate the associations between dietary patterns and CBF risk in a population living in a coal-burning fluorosis area. We extracted three distinct dietary patterns, which are high protein, healthy and easy-to-roast. We found that the healthy dietary pattern had a significant protective effect against CBF, whereas the easy-to-roast dietary pattern was related to an increased risk of CBF.

In present study, we found that a healthy dietary pattern, typified by a high intake of fruit, dairy, legumes, nuts and fungus, was associated with a decreased risk of CBF. Although no studies specifically reported the association between bone damage and CBF, there were many studies that explored the relationship between dietary pattern and bone health. A similar pattern was observed in a matched case-control study of hip fractures in a Chinese population, where a healthy dietary pattern of nuts, fungus, soy and milk were found to be beneficial to bone health (20). Recently, two meta-analyses assessed the relationship between dietary patterns, bone mineral density and the risk of fracture and suggested a healthy diet reduced risk of low BMD in different age groups. The studies also compared the risk of fracture between high and low food intake categories of healthy diet patients (OR = 0.81; 95% CI: 0.69, 0.95; *p* = 0.01) (21, 22). The studies indicated a healthy diet was characterized by “Calcium foods,” “Dairy and whole grains,” “Fruit, milk, and whole grains,” “Milk and root vegetables,” “Vegetable-fruit-soy,” and “Dairy-fruit” (21, 22). In the present study, we found similar components to these healthy dietary patterns. Therefore, healthy diet had a beneficial effect on CBF in the present study and may be attributed to its protective role in bone health. The observed healthy dietary pattern has a balanced food composition, which may explain its beneficial effect against CBF. Additionally, Food choices may be influenced by educational level of participant. Participants with higher education were more likely to follow a healthy diet because they had access to healthy food items. No epidemiological studies have explored the association between nuts, mushrooms, and fluorosis, although a few studies have found that dairy and fruit have protective effects against fluorosis (23–25). Patients who adhered to a healthy dietary pattern had a high intake of calcium, magnesium, carotenoids, vitamin C, vitamin E, and isoflavones. Experimental studies have demonstrated that dietary calcium intake promotes the formation of a high bone formation and adequate dietary calcium or calcium supplements may contribute to a lower risk of fluorosis as calcium antagonizes the effects of fluoride (26, 27). Additionally, oxidative stress is a prime contributing mechanism in CDF and antioxidative nutrients may play important role in preventing CDF (28). It was reported that lycopene combated NaF-induced apoptosis of ameloblast cells and dental fluorosis by improving oxidative stress and down-regulating the caspase pathway (29). Furthermore, vitamin E and lycopene have been shown to prevent fluorosis-induced spermatogenic cell apoptosis through the suppression of oxidative stress-mediated JNK and ERK signaling pathways (30). Several studies suggested isoflavones from legumes and vitamin C may prevent fluorosis by antagonizing the oxidative stress damage caused by fluorine (31, 32). Therefore, we speculate that the antioxidant effects and effect of calcium on bone metabolism may partly explain the protective effects of healthy diets.

This analysis noted a positive association between the easy-to-roast food dietary pattern and CBF risk. We compared the factor scores of easy-to-roast dietary patterns between two groups and found high scoring individuals had a preference for roasted foods. The stratified analysis showed that the positive association between the easy-to-roast foods and fluorosis only existed in subjects who like roasting food. These results indicated the cooking method itself may be an actual risk factor for developing CBF. We conducted this case-control study in Zhijin County, where the moist climate requires residents to burn stoves with mixed fluoride-rich coal and clay. Residents were used to roasting foodstuff over it, such as corns, potato, squash and red chili. Cured meat is also a roasted food and residents commonly hang it for more than 2 months in a house polluted by fluoride dust in Zhijin County. Thus, this dietary pattern involved foods which were easy to roast before consumption. The combustion process released fluoride from the coal and fluorine content significantly increases on the roasted foodstuffs. Previous studies reported fluoride contents of roasted chili, corn, squash, potatoes and cured meat reached 419.8 mg/kg (33), 39.5 mg/kg (33), 121.8 mg/kg (34), 134.6 mg/kg (35), and 182.2 mg/kg (36), respectively. Corn and potatoes are the primary foods consumed by residents of Zhijin and daily mean fluoride intake (per person) from corn was 37.6 and 3.5 mg from potatoes (37). Previous studies demonstrated that particular roasted foods, such as corn, chilli, and potatoes, were the primary pathological cause of coal-burning fluorosis, because of the fluorine pollution coming from the coal briquettes and binder clay (6, 37, 38). Therefore, the present study suggests that the easy-to-roast dietary pattern in our study was positively associated with a risk of fluorosis not because the foods themselves were a source of fluorine, but rather roasting food over coal stoves caused the fluorine pollution.

In subgroup analysis, we observed a protective effect of the healthy dietary pattern against fluorosis in women but not in men. The gender difference in the association between dietary pattern and fluorosis has not been clearly explained. Diet might influence fluorosis between men and women differently due to sex hormones, or sex-specific genes, which are related to the control of fluorosis. Additionally, one of the main foods in this healthy dietary pattern were legumes, which contain the phytoestrogen isoflavone. Meta-analysis and randomized controlled trials demonstrated soy isoflavones have potential bone-specific effects in women (39, 40). The matrix of soy protein-enriched soy isoflavones may improve their bioavailability and biologic efficacy, improving tibia bone architecture (41). Thus, the negative association between the healthy dietary pattern and CBF in women may be attributed to estrogen's effect on isoflavones. Further studies are needed to clarify underlying mechanisms of this process.

The present study had several limitations. First, given the case-control design, we were unable to evaluate the causal associations between dietary patterns and the risk of fluorosis, which need to be confirmed in future studies. Second, it is known that FFQs have measurement error to some extent, although the FFQ used in this study has been validated (16). Third, the factor analysis requires many subjective decisions related to the selection of food groups, including decisions about the number of factors extracted, the type of rotation, and the interpretation and labeling of the factors. Although, this 1:1 matched case-control study was based on community populations, fluorosis and control were not randomly selected, which limited the generalization of findings. Finally, although we adjusted many factors for the statistical analysis, residual confounding was still unavoidable due to potential measurement errors and missing adjustments for some unmeasured factors.

In summary, the present study identified that a healthy dietary pattern has a protective effect against CBF, and that an easy-to-roast dietary pattern was significantly associated with an increased risk of CBF. Our findings suggest that a high consumption of fruit, dairy, legumes, nuts, mushrooms, and a low consumption of roasted corn, potatoes, chillis, and squash may protect against CBF in populations living in coal-burning fluorosis areas.

## Data Availability Statement

The datasets generated for this study are available on request to the corresponding author.

## Ethics Statement

The studies involving human participants were reviewed and approved by Medical Ethics Committee of Zunyi Medical University. The patients/participants provided their written informed consent to participate in this study.

## Author Contributions

JL designed the study, and conducted the study, and revised the manuscript. SY and ML was responsible for the data management, analysis, and wrote the first draft of the manuscript. XM critically revised the manuscript. NT, XZ, and TC assisted in conducting the research and data collection. DW developed the idea and proofread the manuscript.

### Conflict of Interest

The authors declare that the research was conducted in the absence of any commercial or financial relationships that could be construed as a potential conflict of interest.
