# Supplementary material for: Association Between Dietary Patterns and Fluorosis in Guizhou, China
Source: Front Nutr. 2020 Jan 21;6:189. doi: 10.3389/fnut.2019.00189 (PMC6985547; doi:10.3389/fnut.2019.00189)
Supplement: Supplementary file 1 [file Data_Sheet_1.PDF]

Supplementary Table

Supplementary Table 1 Multivariate-adjusted OR (95% CIs) of fluorosis for each tertile of dietary pattern by subgroups of gender and whether or not like roasting food

	Tertiles of factor scores for dietary pattern			*P*-trend	*P-interaction*
	Tertile 1§	Tertile 2	Tertile 3		
High protein pattern					0.615
man	1	0.86（0.36～2.04）	1.09（0.47～2.56）	0.786	
women	1	2.49（1.21～5.14）	1.65（0.71～3.87）	0.185	
Prudent pattern					0.034
man	1	0.72（0.34～1.49）	0.76（0.34～1.71）	0.448	
women	1	0.22（0.10～0.52）	0.22（0.11～0.52）	＜0.001	
Easy-to-roast pattern					0.101
man	1	1.66（0.66～4.20）	5.05（1.92～13.28）	0.004	
women	1	1.82（0.86～3.86）	1.34（0.61～2.95）	0.522	
High protein pattern					0.263
like roasting food	1	1.06（0.53～2.13）	1.44（0.65～3.18）	0.380	
Not like roasting food	1	2.38（1.00～5.62）	1.01（0.42～2.47）	0.987	
Prudent pattern					0.355
like roasting food	1	0.23（0.10～0.53）	0.29（0.13～0.63）	0.002	
Not like roasting food	1	0.66（0.31～1.43）	0.36（0.15～0.88）	0.023	
Easy-to-roast pattern					0.043
like roasting food	1	2.89（1.26～6.62）	2.44（1.13～5.33）	0.033	
Not like roasting food	1	1.10（0.46～2.63）	1.52（0.64～1.24）	0.153	

Note. Abbreviations：*OR*, odds ratio；*CI*, confidence interval. §Tertile1 was the reference tertile. Model 1 Crude adjusted ORs were obtained without further adjustment of covariates. Model 2 adjusted for duration of residence in zhijin, ethnic, marital status, education level, body mass index, urinary fluoride level, household income, smoking, alcohol drinking, tea drinking, fuel type, improved stove use, roasting food
